# The association between passive social media use and personal growth initiative: a moderated mediation model

**DOI:** 10.3389/fpsyg.2026.1668890

**Published:** 2026-04-09

**Authors:** Heng Yue, Yin Bai, Shiwen Gao, Min Liu, Xuemin Zhang

**Affiliations:** 1 College of Physical Education, Inner Mongolia Normal University, Hohhot, China; 2 School of Journalism and Communication, Xiamen University, Xiamen, China; 3 School of Physical Education, XingAn Vocational and Technical College, Ulanhot, China; 4 College of Business and Public Management, Wenzhou-Kean University, Wenzhou, China; 5 College of Psychology, Inner Mongolia Normal University, Hohhot, China; 6 School of Nursing, Baotou Medical College, Baotou, China

**Keywords:** passive social media use, personal growth initiative, self-control, physical activity, moderated mediation model

## Abstract

**Introduction:**

Most of the previous studies have investigated the effect of personal growth initiative on college students’ positive development by using it as a predictor. However, factors that may impact personal growth initiative have not been widely investigated; the underlying mechanism is still unclear.

**Methods:**

By establishing a moderated mediation model, the current study examined the relationship between passive social media use and personal growth initiative as well as the mediating role of self-control and the moderating role of physical activity. A paper-pencil-based survey was performed; the final sample included 476 participants (263 females).

**Results:**

The results demonstrated that passive social media use was negatively correlated with personal growth initiative, and this link is mediated by self-control. Physical activity moderated the impacts of passive social media use on both personal growth initiative and self-control, indicating that the two associations were stronger for individuals with high physical activity levels than for those with low physical activity levels.

**Discussion:**

These results were consistent with the strength model of self-control and, meanwhile, indicated that participating in physical exercise was helpful for buffering the negative effects of passive social media usage on self-control and personal growth initiative. The current study may be conducive to understanding the associations among the study variables, taking effective and suitable measures to improve personal growth initiative, and finally advancing individuals’ positive development.

## 1 Introduction

Personal growth initiative is defined as a series of cognitive and behavioral skills that are significant for self-improvement (Robitschek et al., 2012); some scholars also consider it a valuable resource that endows college students to accomplish positive development and facilitate personal growth (Weigold and Robitschek, 2011). Previous studies have found that personal growth initiative can promote the fulfillment of the basic psychological needs such as autonomy, competence, and relatedness (Weigold et al., 2021); therefore, it can protect college students from depression, anxiety, and other psychological distresses (Shigemoto et al., 2025; Weigold et al., 2020); buffer against suicide ideation and addictive behaviors (Robitschek et al., 2022; Yue et al., 2025); promote positive coping styles and, meanwhile, resist negative coping styles (Weigold et al., 2024); and enhance the levels of academic performance, hope, well-being, and life meaning (Kong et al., 2025; Li et al., 2024). Although personal growth initiative has so many benefits for college students, its antecedents have not been widely explored. In fact, only a few studies have investigated its positive antecedents; the negative predictors of personal growth initiative remain unclear. Therefore, researching and eliminating the risk factors of positive personal development and cultivating personal growth initiative among college students have become important concerns within the area of higher education.

Nowadays, in the digital age, the influence of social media on people has been a hot topic among both social scientists and the general public. Although social media brings various conveniences that facilitate our daily lives, the negative impacts that result from problematic usage habits should also be held in high regard. In fact, previous studies have found that passive social media use has caused many detrimental consequences; for instance, upward social comparison during passive social media use can cause negative emotions such as depression and envy; browsing social media before sleeping often leads to low sleep efficiency; poor academic performance is often observed in students who spend large amounts of time and effort on passively using social media; and interpersonal problems are also frequently caused by lacking social skills; people who passively indulge themselves in the virtual world often lack the basic social skills and have weak and low interpersonal relationships (Yue et al., 2022; Zhang et al., 2020). In this way, passive social media use may decrease personal growth initiative. As most of the previous studies were conducted by regarding personal growth initiative as one of the protective factors of problematic social media use, the influence of the latter on the former has not been considered. Thus, the present study aims to examine the impact of passive social media use on personal growth initiative and the underlying psychological mechanism. The results of this study may be conducive to understanding the associations between the study variables, taking effective and suitable measures to improve personal growth initiative, and finally advancing individuals’ positive development.

### 1.1 The association between passive social media use and personal growth initiative

Passive social media use refers to the behavior that individuals merely browse the content created and posted on social media by others without interactions with the owner (Verduyn et al., 2017). Passive social media use is also known as lurking, which is defined as browsing social media content with no direct exchanges (Amichai-Hamburger et al., 2016; Gazit et al., 2018). Although, to the best of our knowledge, there is no research that has directly investigated the association between passive social media use and personal growth initiative, some findings provide useful information that may be helpful for understanding the relationship between the two study variables. Previous scholars indicated that social media addiction is a detrimental consequence of passive social media use and, meanwhile, a significant antecedent of personal growth initiative (Brailovskaia and Margraf, 2022; Yue et al., 2025). Researchers suggested that individuals, especially the passive users, who spend a lot of time on the social media platforms or applications may have little time and effort to improve themselves and may neglect various opportunities for personal development (Hall and Liu, 2022; Roberts and David, 2023). Some studies have also confirmed that passive social media use brings about various psychological distresses such as depression, stress, loneliness, and so on (Zhang et al., 2020). These negative ramifications will limit the users’ horizons, reduce their self-efficacy, and hinder their intentions and behaviors to better themselves. Besides, the control-value theory posits that individuals’ emotions can significantly impact their activity achievements, and negative experiences often lead to failures in learning (Pekrun, 2024). Since negative rather than positive information spreads more rapidly and widely on social media (Tsugawa and Ohsaki, 2015), and personal growth initiative is a set of cognitive and behavioral skills that need to be learned, therefore, people who browse social media more frequently may be more impacted by the negative messages such as the news about unemployment, qualification inflation, social injustice, and so on. According to the control-value theory, this will hinder their abilities to learn the personal growth skills. Based on the findings and considerations of prior researchers, the present study hypothesizes that passive social media use is negatively correlated to personal growth initiative.

### 1.2 The mediating effect of self-control

Previous studies have found that passive social media use is negatively correlated with self-control, and it can significantly and positively predict impulsive behavior (Zhang S. et al., 2025; Zhang J.-Y. et al., 2025; Zheng et al., 2024). Some researchers also argued that the addictive nature of social media, such as endless scrolling and instant gratification, may also hurt people’s self-control and goal achievement (Shaji et al., 2023). Besides, prior scholars have suggested that passive social media users often compare themselves with others they regard as superior; this action can bring them various negative emotions such as depression, stress, and social anxiety. Meanwhile, negative news and messages (such as unemployment) contained in social media will also make passive users experience frustration and disappointment; all these impacts can contribute to the reduction of self-control as well (Wang et al., 2025; Zhang et al., 2020). On the one hand, according to social learning theory, people often learn impulsive behaviors through observation, imitation, and reinforcement (Bandura and Walters, 1977). When they see their peers or friends get positive comments, show some beautiful things, or have some novel experiences on social media, they may also have the desire to do so and exhibit impulsive behaviors. In the long run, through positive reinforcement, their self-control capacity will be gradually decreased. On the other hand, the strength model of self-control posits that self-control is a limited resource that can be consumed through attempts to regulate thoughts and manage emotions (Baumeister et al., 2007). Therefore, individuals’ self-control capacity will be depleted through regulating these adverse thoughts and feelings. Consequently, they will have low levels of self-control.

When self-control is impaired by the reinforcement of the impulsive behaviors or the depletion of the self-control resource, individuals often have poor performance in the subsequent tasks, and they may be easily influenced by various temptations and cannot insist on the long-term goals (Baumeister, 2018). Findings of several studies support this viewpoint by showing that self-control mediates the link between trait anxiety and academic achievement (Shi and Ma, 2021), between social exclusion and time discount rate (Yang P. et al., 2024; Yang X. et al., 2024), and between childhood trauma and adolescent game addiction (Zhong et al., 2023). Since personal growth initiative includes a set of behavioral and cognitive skills that need to be acquired through persistent efforts and overcoming various difficulties, individuals whose self-control is decreased will be unlikely to be successful in learning and striving for self-improvements and will not have a high level of personal growth initiative. In fact, prior researchers have indicated that self-control has a direct positive effect on personal growth initiative (Huang, 2022; Tang, 2024). Therefore, the current study assumes that when self-control capability is diminished by lots of problematic behaviors and negative experiences, personal growth initiative will also be decreased.

From what has been mentioned above, it is reasonable to hypothesize that self-control may mediate the link from passive social media use to personal growth initiative.

### 1.3 The moderating effect of physical activity

Previous scholars have shown that by elevating the densities of dopamine, serotonin, and norepinephrine, physical activity can contribute to relieving negative emotions and promoting positive emotions (Li et al., 2022; Voss et al., 2011). Some researchers have also found that physical activities in school or community environments can promote individuals’ interactions with the surrounding interpersonal environment and help them establish positive connections; these benefits are conducive to improving social competence, interpersonal relationships (Hu et al., 2025), and psychological capital (Dong et al., 2023). All these physiological and interpersonal advantages make physical activity a significant factor that can buffer the impacts of problematic mobile phone use on depression (Tao et al., 2020), stressful life events on self-worth (Zheng et al., 2023), time anxiety on sleep quality (Sun L. et al., 2024; Sun Z. et al., 2024), and perceived discrimination on self-esteem and on mental health (Yang P. et al., 2024; Yang X. et al., 2024). Since physical activity may diminish various adverse consequences of passive social media use and may contribute to individuals’ positive development, it is possible that it can moderate the association between passive social media use and personal growth initiative.

Based on the strength model of self-control (Baumeister et al., 2007), prior studies have confirmed that regular physical activity can increase the self-control resource and promote the restoration of this resource (Wei, 2023); some scholars have also demonstrated that physical activity can increase blood flow and oxygen uptake, which will promote the elevation of cognitive control (Zheng et al., 2023). In addition, previous researchers found that executive function, especially the inhibitory control ability, can be enhanced through acute or chronic physical activity (Montalva-Valenzuela et al., 2022). In fact, the positive effect of physical activity on self-control has been confirmed by prior studies (Dong et al., 2023; Du and Zhang, 2022). Combined with the discussion in the prior paragraph, it is reasonable to hypothesize that physical activity can moderate the association between passive social media use and self-control.

### 1.4 The present study

Based on the control-value theory and the strength model of self-control (Baumeister et al., 2007; Pekrun, 2024), passive social media use may bring about various negative emotions and experiences that may (1) contribute to the decrease of acquiring the skills for personal growth and (2) deplete individuals’ self-control capacity. Because previous studies have demonstrated that physical activity can benefit self-control and is positively correlated with personal growth initiative, it may buffer the negative impacts of passive social media use on self-control and personal growth initiative.

**Figure 1:**
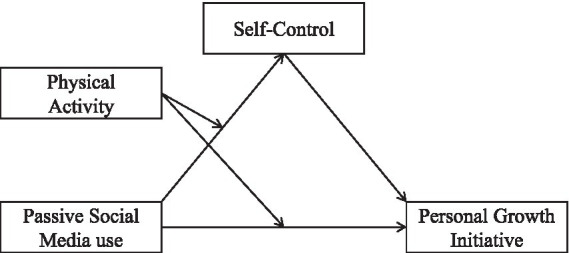
The moderated mediation model.

From what has been mentioned above, a moderated mediation model was established to test the associations between passive social media use and personal growth initiative as well as the underlying psychological mechanism (Figure 1). The main hypotheses were listed as follows: (1) passive social media use and personal growth initiative; (2) self-control mediated the link from passive social media use to personal growth initiative; and (3) physical activity moderated the association from passive social media use to self-control and from passive social media use to personal growth initiative.

## 2 Methods

### 2.1 Participants

The study was approved by the ethics committee of the College of Physical Education, Inner Mongolia Normal University. Participants were recruited from 5 universities in the Inner Mongolia Autonomous Region. A paper-and-pencil-based survey was conducted to collect data. Questionnaires were distributed in the after-school time. When the after-school bell rang, the teacher (researcher) asked the students whether they would like to participate in the current research. Individuals who intended to participate were kept in the classroom, and those who refused to join were allowed to leave the classroom. Next, before sending out the questionnaires, all the students in the classroom were told that the survey was anonymous, the information they provided would be used only for scientific research and would be kept confidential, they could withdraw from the survey at any time they would like, and there would be no adverse impact on them. Then the researcher distributed the questionnaires to the students one by one. Informed consent was presented on the first page of the questionnaire. Participants were asked to read the informed consent before starting to fill out the questionnaires. Their data would be used in the analysis only when they chose the “I agree” option at the end of the informed consent.

Originally, 600 questionnaires were sent out; 563 questionnaires were received. After removing 87 invalid ones, finally, 476 participants’ responses were used in the formal data analyses. There were 213 males and 263 females. The average age of these college students was 20.26 ± 1.66 years old, ranging from 18 to 25 years.

### 2.2 Measurements

#### 2.2.1 Passive social media use

Passive social media use was measured by the surveillance use scale (Tandoc et al., 2015). Since this instrument had been validated in a Chinese context, the Chinese version of the scale was employed (Liu et al., 2017). This instrument consisted of 4 items; participants were asked to rate each item on a 5-point Likert scale ranging from 1 (never) to 5 (very often). Higher sum scores on this scale indicated greater levels of passive social media use. This scale had been used in much previous research (Han et al., 2025; Sun L. et al., 2024; Sun Z. et al., 2024; Zhang and Zeng, 2023). In the present study, the Cronbach’s *α* coefficient of this scale was 0.826.

#### 2.2.2 Personal growth initiative

Personal growth initiative was assessed by the personal growth initiative scale-II (Robitschek et al., 2012). Since this instrument had been validated in a Chinese context, the Chinese version of the scale was employed (Xu et al., 2019). This scale included 16 items rated on a 6-point Likert scale (0 = Disagree Strongly to 5 = Agree Strongly). A higher total score on this scale indicated a higher degree of personal growth initiative. This scale had been used in much previous research (Robitschek et al., 2022; Seidman et al., 2022; Yue et al., 2025). In the current study, the Cronbach’s *α* coefficient of this scale was 0.900.

#### 2.2.3 Self-control

Self-control was measured by the brief self-control scale (Morean et al., 2014). This scale contained 7 items. All the items were scored on a 5-point Likert scale ranging from 1 (not at all) to 5 (very much), except for 4 items that were reverse-scored. Higher total scores on this scale suggested higher levels of self-control. This scale had been validated and used in previous research (Wang, 2025; Yue et al., 2022). In the present study, the Cronbach’s α coefficient of this scale was 0.721.

#### 2.2.4 Physical activity

Physical activity was assessed by the physical activity rating scale-3 (Liang and Liu, 1994). This scale had 3 items assessing the intensity, frequency, and duration of physical activity. These items were rated on a 5-point scale. The total score is calculated by using the equation: intensity × frequency × duration. A higher total score suggested a higher level of physical activity. This scale had been validated and used in previous research (Gong et al., 2023; Ke et al., 2024; Zhu et al., 2024). In the present study, the Cronbach’s α coefficient of this scale was 0.642.

### 2.3 Statistical analyses

Descriptive statistics and correlation analyses were conducted by using SPSS; the mediating effect of self-control and the moderating effect of physical activity were examined by employing the SPSS Process Macro (Models 4 and 8). To assess the statistical significance of the mediation and the moderation effects, the bootstrap method with 5,000 resamples was used to evaluate the 95% bias-corrected confidence intervals of the conditional direct and indirect effects. If the 95% confidence interval did not include zero, the effect was considered significant.

## 3 Results

### 3.1 Descriptive statistics

The results of the descriptive statistics and correlation analyses were presented in Table 1. Passive social media use was negatively correlated with self-control (*r* = −0.291, *p* < 0.01), personal growth initiative (*r* = −0.404, *p* < 0.01), and physical activity (*r* = −0.164, *p* < 0.01). These results meant that passive social media users were more likely to have lower levels of self-control, physical activity, and personal growth initiative. Self-control was positively correlated with physical activity (*r* = 0.471, *p* < 0.01) and personal growth initiative (*r* = 0.367, *p* < 0.01). That is, people with high levels of self-control tend to have high levels of physical activity and personal growth initiative. Personal growth initiative was positively associated with physical activity (*r* = 0.308, *p* < 0.01). This indicated that students with high levels of physical activity were more likely to have high levels of personal growth initiative than those with low levels of physical activity.

**Table 1:** Descriptive statistics and correlation analyses.

Variables	*M*	*SD*	1	2	3	4
1. PSU	11.08	3.63	1			
2. SC	23.07	4.05	−0.291**	1		
3. PA	19.46	22.05	−0.164**	0.471**	1	
4. PGI	59.78	15.60	−0.404**	0.367**	0.308**	1

PSU, Passive social media use. SC, Self-Control. PGI, Personal Growth Initiative, PA, Physical Activity. **p < 0.01.

### 3.2 Common method bias test

To detect the possible common method bias, the Harman’s single-factor test was performed, and the results demonstrated that the first extracted factor explained 26.73% of the variance of all items, and this value was smaller than the 40% threshold (Podsakoff et al., 2003), indicating that there is no serious common method bias in the current study.

### 3.3 The mediating effect of self-control

By using the PROCESS macro (Model 4), the mediating effect of self-control was tested. The results suggested that passive social media use was negatively correlated with personal growth initiative (*b* = −1.400, *p* < 0.01) and self-control (*b* = −0.325, *p* < 0.01), indicating that higher levels of passive social media use could significantly and negatively predict personal growth initiative and self-control. Self-control was positively associated with personal growth initiative (*b* = 1.048, *p* < 0.01), demonstrating that higher levels of self-control could significantly and positively predict personal growth initiative. The direct effect of passive social media use on personal growth initiative was significant (*b* = −1.400, 95% CI = [−1.754, −1.042]), and the indirect effect of self-control was significant as well (*b* = −0.341, 95% CI = [−0.490, −0.212]). Thus, self-control partially mediated the link between passive social media use and personal growth initiative, indicating that passive social media use could directly predict personal growth initiative and could indirectly predict personal growth initiative through self-control (Table 2).

**Table 2:** The results of the mediating effect of self-control.

Effect	*b*	95%CI	
		Lower	Upper
Direct effect			
PSU → PGI	−1.398	−1.754	−1.042
PSU → SC	−0.325	−0.421	−0.229
SC → PGI	1.0480	0.729	1.367
Indirect effect			
PSU → SC → PGI	−0.341	−0.490	−0.212

PSU, Passive social media use. SC, Self-control. PGI, Personal growth initiative, PA, Physical activity. **p < 0.01.

### 3.4 The moderating effects of self-control

By employing the PROCESS macro (Model 8), the moderating effect of self-control in the associations between passive social media use and self-control and the link between passive social media use and personal growth initiative were examined. The results were displayed in Table 3.

**Table 3:** The results of the moderated mediation model.

Effect	*b*	95%CI	
		Lower	Upper
Outcome variable: self-control			
Age	0.123	−0.724	0.318
Gender	0.483	−0.166	1.132
PSU	−0.345**	−0.461	−0.229
PA	0.031	−0.110	0.073
PSU x PA	0.005*	0.001	0.008
Outcome Variable: Personal growth initiative			
Age	0.357	−0.409	1.1229
Gender	−0.6343	−3.180	1.912
PSU	−1.709**	−2.180	−1.238
SC	0.715**	0.361	1.070
PA	−0.042	−0.206	0.122
PSU × PA	0.015**	0.001	0.030
Conditional indirect effect(s) of PSU on PGI			
M – 1 SD	−0.244	−0.391	−0.114
M + 1 SD	−0.113	−0.239	−0.018

PSU, Passive social media use. SC, Self-control. PGI, Personal growth initiative, PA, Physical activity. *p < 0.05; **p < 0.01.

**Figure 2:**
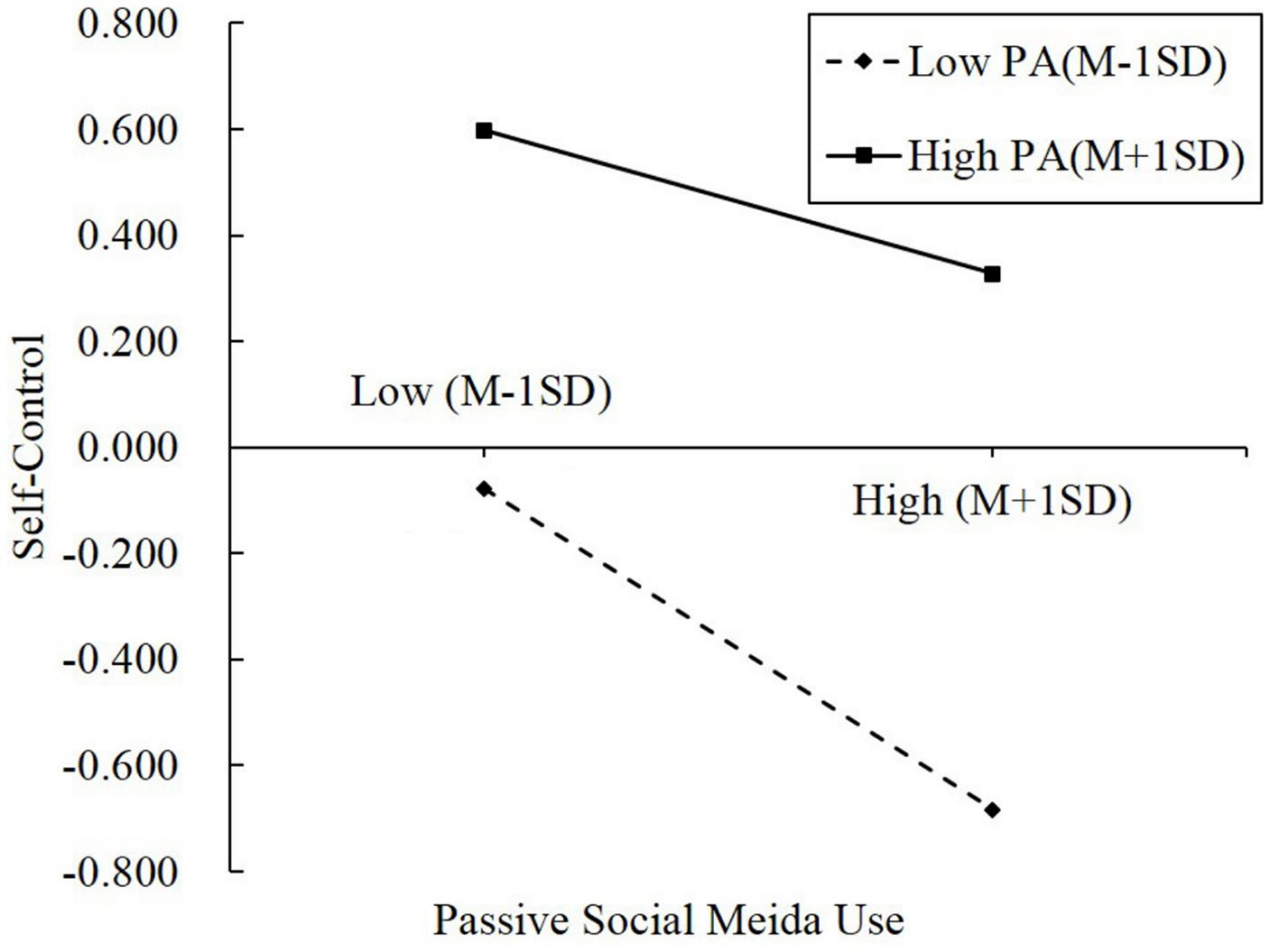
The relationship between passive social media use and self-control at two levels of physical activity.

After controlling for gender and age, passive social media use was negatively associated with self-control (*b* = −0.345, *p* < 0.01), and this effect was moderated by physical activity (*b* = 0.005, *p* < 0.05, Δ*R*^2^ = 0.009). The plot of the association between passive social media use and self-control at two levels of physical activity (M – 1 SD and M + 1 SD) was presented in Figure 2. The negative association between passive social media use and self-control was weaker for individuals with high physical activity (1 SD above the mean) than for individuals with low physical activity (1 SD below the mean).

**Figure 3:**
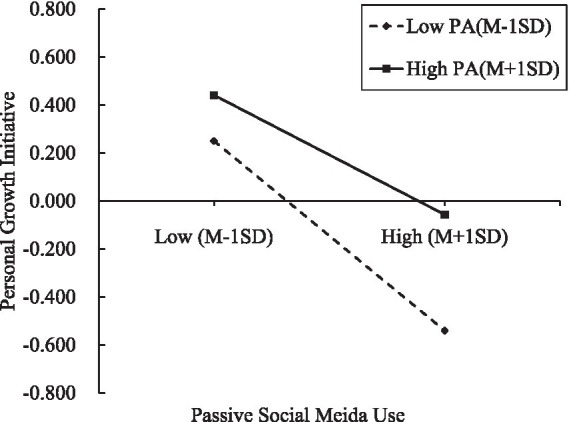
The relationship between passive social media use and personal growth initiative at two levels of physical activity.

The results further indicated that personal growth initiative was significantly predicted by both passive social media use (*b* = −1.709, *p* < 0.01) and self-control (*b* = 0.715, *p* < 0.01); the effect of passive social media use on personal growth initiative was moderated by physical activity (*b* = 0.015, *p* < 0.01). The figure of the association between passive social media use and personal growth initiative at two levels of physical activity (M – 1 SD and M + 1 SD) was presented in Figure 3. The negative association between passive social media use and personal growth initiative was weaker for individuals with high physical activity (1 SD above the mean) than for individuals with low physical activity (1 SD below the mean).

## 4 Discussion

By employing the SPSS Process Macro (Models 4 and 8), the current study explored the associations between passive social media use, self-control, personal growth initiative, and physical activity. The main findings and implications are presented below.

### 4.1 The association between passive social media use and personal growth initiative

The results of the data analyses showed that passive social media use was negatively linked to personal growth initiative. The reason may lie in the following facts. Firstly, passive social media users often browse numerous online content, such as short videos and posts, absentmindedly and with high frequency. On the one hand, constantly dealing with fragmented information will cause the brain to become accustomed to immediate gratification and rapid switching, which makes it intolerant of the growth process that requires a long-term investment (such as learning a language or mastering a skill) to achieve rewards (Li et al., 2025). On the other hand, passively accepting ready-made viewpoints will reduce the opportunities for independent thinking and problem-solving, thereby weakening the metacognitive abilities necessary for personal growth. Secondly, social media users are often inevitably impacted by the recommendation algorithms that can infinitely prolong the users’ residence time. In this way, their time that could have been used for learning skills and exercise will be consumed by endless social media use (Hall and Liu, 2022; Kraut et al., 1998), and meanwhile, a large amount of information will also occupy the users’ working memory, making them feel mentally exhausted and leaving them with no energy to think and plan their personal growth. At last, personal growth initiative requires the users to be screenwriters of their own lives, while passive social media use makes them observers of others’ lives. In this way, compared with normal users, individuals who are high in passive social media use will have fewer chances to figure out their personal growth directions and goals, and their social values and social behaviors will be distorted. Such as by upward social comparison, they will get more anxiety, depression, learned helplessness, and materialism (Chen and Pu, 2022; Jameel et al., 2024; Son and Heo, 2020). According to the control-value theory, these adverse experiences and undesirable value orientations will negatively impact students’ acquisition of the personal growth skills (Pekrun, 2024). From what has been mentioned above, the association between passive social media use can be understood.

### 4.2 The mediating effect of self-control

In the current study, passive social media use was negatively correlated with self-control. This result contributes to the literature by suggesting that self-control can be decreased by passive social media use, and it was partially in line with previous studies indicating that passive social network site usage is negatively correlated with self-control, and it can positively predict compulsive behavior (Chen et al., 2022; Zheng et al., 2024). This may be because, according to the Zeigarnik effect (Zeigarnik, 1938), unfinished or interrupted tasks ‌often trigger psychological tension, which people seek to resolve by reaching closure. In fact, the fragmented nature of social media information is just like the incomplete cognitive tasks, which the passive users seek to achieve, consciously or unconsciously, through endlessly scrolling (Zeba et al., 2026). In the long run, these compulsive usage habits will decrease their self-control capacities. Secondly, according to the stimulus-organism-response framework, social media’s unique features may deprive the possibility of self-control. Algorithmic personalization and infinite scrolling often make users exposure to repetitive triggers (Park and Jung, 2024). In this way, endlessly scrolling will take the place of deeply thinking; high frequency and intensity of stimulations make it hard for the users to generate self-control intentions, which may decrease their self-control levels. Thirdly, by engaging in the posters’ narratives, passive users’ mental energy will be consumed by empathizing, evaluating, and identifying with the stories of others (Tyrväinen et al., 2025), and they will also experience envy, loneliness, stress, depression, and other negative emotions through upward social comparison (Li and Ma, 2019; Wang et al., 2019; Zhang et al., 2020). According to the strength model of self-control (Baumeister et al., 2007), self-control resources can be depleted through efforts to regulate thoughts, manage emotions, and resist various temptations, and the lack of rest (sleep) will hinder people from recovering from the depleted status. Therefore, the detrimental consequences resulting from passive social media use can reduce people’s self-control capacities.

In terms of the association between self-control and personal growth initiative, researchers have found that previous exertion of self-control may undermine the performance that requires self-control in the consecutive tasks; this will not only decrease the motivation and persistence but also increase the possibility of failure in attaining the preset goals (Chow et al., 2015). Thus, when individuals’ self-control capacities are depleted by numerous negative outcomes originating from passive social media use, they may have low levels of personal growth initiative. Besides, previous scholars have demonstrated that self-control completely mediates the association between the functional connectivity between the left dorsolateral prefrontal cortex and the right inferior temporal gyrus and achievement motivation (Yang et al., 2025). That is to say, a low level of self-control often originates from a weak functional connectivity between the two brain areas, and this will further decrease their motivations to make plans and learn the skills for active self-improvement. In addition, according to the regulatory focus theory, there are two cognitive–motivational systems that direct individuals’ goal pursuit behaviors – the promotion system and the prevention system (Higgins, 1997). Promotion-focused individuals are often preoccupied with pursuing ideals, hopes, and achievements, while a prevention-focused person often takes the avoidance goal pursuit strategies and engages in vigilance, responsibilities, and ought. Previous studies have verified that people with low self-control tend to adopt prevention-focus orientation (Cheung et al., 2014). This defensive strategy will hinder them from taking actions to improve themselves, manifesting as low personal growth initiative.

### 4.3 The moderating effect of physical activity

The results of the current study indicated that physical activity moderated the links from passive social media use to self-control and to personal growth initiative. This implies that passive social media use with higher physical activity is less likely to have lower levels of self-control and personal growth initiative. In terms of self-control, this may be because by facilitating the efficient activation of the prefrontal cortex, stimulating neuron growth, and consolidating synaptic connections, physical activity can effectively promote the improvement of executive function, especially inhibitory control (Matrisciano et al., 2025); and by altering the brain’s structure and function and increasing levels of dopamine, serotonin, and norepinephrine, physical activity can contribute to the relief of depression, stress, loneliness, and other negative emotions (Wang et al., 2024). In this way, individuals’ self-control capacities will be improved, and the impact of passive social media use on self-control will be buffered. Besides, physical activities provide a sense of competence through skill improvement, an experience of belonging through team participation, and a feeling of autonomy through making their own decisions (Xu et al., 2021). The satisfaction of the basic psychological needs will also decrease individuals’ dependence on social media and promote the enhancement of self-control (Gilbert et al., 2023; Zhang S. et al., 2025; Zhang J.-Y. et al., 2025). In addition, through physical activities, an individual’s evaluation criteria will be transformed from upward social comparison to self-reference (today I am better than yesterday’s me) (Liu and Miao, 2024). The shift of the evaluation framework will reduce the sensitivity and reliance on social comparisons, and in this way, the negative effects of passive social media uses (such as social comparison) on self-control will also be decreased. From what has been mentioned above, the buffering effect of physical activity on the link from passive social media to self-control can be understood.

As far as personal growth initiative is concerned, just as what has been mentioned above, while passive social media use places the users in the position of “spectators of others’ stories,” physical activity will reposition them as “the authors of their own stories” (Xu, 2025). Through physical exercise, people may have more subjective consciousness, accept themselves, like themselves, and become stronger and more energetic to cope with various tasks in their daily lives (Xu, 2025). In this way, they will gradually engage in the process of positive self-development. Besides, according to the identity theory, when a person’s actions are consistent with their identity, their actions will become more persistent and proactive (Ren et al., 2024; Stets and Burke, 2000). Therefore, through participating in physical exercise, individuals’ identity narratives (I’m an active, optimistic, and high-self-control person) will be gradually formed, strengthened, and internalized, and this identity will also promote their personal growth motivations and behaviors. In addition, some previous studies have indicated that individuals with high physical activity tend to have higher quality interpersonal relationships and be trusted by people around them (Chen et al., 2020; Qin and Dong, 2017); it can enhance participants’ self-esteem, self-efficacy, achievement motivation, and life satisfaction (Cao et al., 2025). Therefore, by offering social resources and boosting the intentions and behaviors of self-improvement, physical activities may contribute to individuals’ positive personal growth and may buffer the negative influence of passive social media use on personal growth initiative. Specifically, because compared with low- or high-intensity exercises, many previous studies have consistently suggested that regular (about 3 times per week) exercise with moderate intensity often has the best effects on people’s self-control and mental health (Herbert, 2022; Nakagawa et al., 2020; Xu et al., 2022). Therefore, in the current study, regular moderate-intensity exercises were suggested for college students.

## 5 Conclusion

The current study explored the association between passive social media use and personal growth initiative. The results suggested that passive social media use was negatively linked to personal growth initiative; this association was mediated by self-control. Besides, physical activity moderated the detrimental influences of passive social media use on self-control and personal growth initiative. Based on the findings of the present study, social media users are encouraged to decrease the time and intensity of passive social media use and actively participate in physical activities, especially regular moderate-intensity exercises, which may be conducive for enhancing the strength of self-control and facilitating the development of personal growth initiative.

## 6 Implications

Firstly, this study showed that passive social media use had an adverse effect on students’ personal growth initiative. This may be because passive social media use can lead to addictive use habits, which take over students’ time and efforts for actively personal growth, or continually browsing the negative information that is delivered by social media makes college students frustrated about their daily lives and disappointed about their future. Therefore, encouraging students to reduce their social media usage time and intensity may be an effective way to promote their positive development.

Secondly, the results indicate that passively browsing social media may hurt students’ self-control. Whether this result comes from the reinforcement mechanisms of media platforms or the negative emotions elicited by the undesirable information delivered by social media, it indeed enlightens us that decreasing the “passive media time” may protect our self-control capacities. Since social media has been widespread in our daily lives, these results imply college students should use this platform temperately so that it can play a constructive rather than destructive role. Besides, this result also demonstrates that self-control is vulnerable to various life events such as passive social media use. Thus, taking measures to avoid the unnecessary trifles and the undesirable information that may deplete our energy may benefit the success in the right things that we must face in daily life.

At last, although physical activity significantly buffered the negative effects of passive social media use on self-control and personal growth initiative, the former was still negatively correlated with the latter, even at the high level of physical activity. This may be because college students often spend more time on browsing social media rather than on exercise. Therefore, encouraging students to participate in regular moderate-intensity physical activity, and meanwhile taking measures to reduce their social media usage time and intensity, would be more effective for their positive personal growth.

## 7 Limitations and recommendations

Firstly, in the current study, all variables were evaluated based on self-reported data. Future research is encouraged to examine these results by using objective data, such as measuring passive social media use by means of social media use time and using accelerometers to measure participants’ physical activity levels. Secondly, this study was conducted by using a cross-sectional design; this may limit causal inference. Thus, longitudinal design is suggested to be employed to test the robustness of these results in future research. At last, participants in this study were college students; this may limit the generalization of the results. Future researchers are recommended to use other samples, such as students in primary school or middle school; this may be helpful for examining and generalizing the results in the present study.

## Data Availability

The raw data supporting the conclusions of this article will be made available by the authors, without undue reservation.
